# Advances in neural architecture search

**DOI:** 10.1093/nsr/nwae282

**Published:** 2024-08-23

**Authors:** Xin Wang, Wenwu Zhu

**Affiliations:** Department of Computer Science and Technology, Beijing National Research Center for Information Science and Technology, Tsinghua University, Beijing 100084, China; Department of Computer Science and Technology, Beijing National Research Center for Information Science and Technology, Tsinghua University, Beijing 100084, China

**Keywords:** machine learning, artificial intelligence, neural architecture search

## Abstract

Automated machine learning (AutoML) has achieved remarkable success in automating the non-trivial process of designing machine learning models. Among the focal areas of AutoML, neural architecture search (NAS) stands out, aiming to systematically explore the complex architecture space to discover the optimal neural architecture configurations without intensive manual interventions. NAS has demonstrated its capability of dramatic performance improvement across a large number of real-world tasks. The core components in NAS methodologies normally include (i) defining the appropriate search space, (ii) designing the right search strategy and (iii) developing the effective evaluation mechanism. Although early NAS endeavors are characterized via groundbreaking architecture designs, the imposed exorbitant computational demands prompt a shift towards more efficient paradigms such as weight sharing and evaluation estimation, etc. Concurrently, the introduction of specialized benchmarks has paved the way for standardized comparisons of NAS techniques. Notably, the adaptability of NAS is evidenced by its capability of extending to diverse datasets, including graphs, tabular data and videos, etc., each of which requires a tailored configuration. This paper delves into the multifaceted aspects of NAS, elaborating on its recent advances, applications, tools, benchmarks and prospective research directions.

## Teaser

A comprehensive survey, vision and view article on the recent advances in neural architecture search techniques.

## INTRODUCTION

Automated machine learning (AutoML) aims to automate the process of developing and deploying machine learning models [1–3]. Since AutoML is able to achieve or surpass human-level performance with little human guidance, it has gained tremendous attention and has been widely applied to numerous areas. A complete AutoML pipeline involves various stages of machine learning (ML), including data preparation, feature engineering, model configuration, performance evaluation, etc. The most widely studied research interests in AutoML are hyperparameter optimization (HPO) [4–8] and neural architecture search (NAS) [9–15]—the former is a well-documented classic topic focusing on the hyperparameter configuration, while the latter is a recent topic concentrating on architecture customization. In this paper, we mainly explore the development and advancement of NAS, which has long been a challenging and trending topic.

In general, NAS plays a crucial role in discovering the optimal neural architecture automatically, saving human efforts from manual design. Since being first proposed by Zoph *et al.* [16], NAS has achieved excellent performances on various tasks, including image classification [17–19], object detection [20–22], semantic segmentation [23–26], text representation [27], graph learning [28,29], neural machine translation [30], language modeling [16,31,32], etc. NAS methods can generally be classified based on tailored designs from the following aspects [9]: (i) search space, (ii) search strategy and (iii) evaluation strategy. In particular, the search space can be further categorized into two types: (1) macro space for the entire network and (2) micro space for modules or blocks of the neural network, where the choices for operators will be determined by the given data. For example, a *convolution* operator may be best suitable for image data, an *attention* operator can be the best fit for sequence data, an *aggregation* operator tends to find its most appropriate position for graph data, etc. The search strategy is used to discover the optimal architecture from the search space, which needs to balance the effectiveness and efficiency simultaneously. Take the following two representative approaches as an example: reinforcement learning (RL) chooses operators based on the potential performance gain and the evolutionary algorithm (EA) selects architectures via simulating the process of biological evolution. The evaluation strategy decides how to estimate the performance of different architectures. For instance, we can utilize multiple trials of training from scratch to access architectures stably and accurately at the cost of a huge amount of computation, as well as employ the family of supernet-based methods to approximately estimate performances with greatly reduced training resources.

The research focus of NAS has been constantly changing and developing over time. In the beginning, it put emphasis on automatic architecture design and outstanding performance [16]. With the help of RL, the family of NAS approaches manages to find good architectures for various multimedia data, including images [33], texts [34], videos [35] and tabulars [36]. However, the computational cost of NAS is extremely expensive for most scenarios, which motivates the devotion of later works to reducing the cost, resulting in the emergence of different strategies such as weight sharing [31,32], evaluation estimation [37,38], etc. Meanwhile, relevant benchmarks [29,39–41] have been published for time-saving, convenient and fair comparison of various NAS algorithms. As a growing amount of attention has been given to NAS, the adaptations of NAS to new problems and data, e.g. graphs [28,42], become the cutting-edge topics. The works focusing on the above new problems have pushed forward to research for tailored designs of search spaces, search strategies and evaluation strategies for various important and trending problems, thus popularizing NAS in more areas.

**Figure 1.**
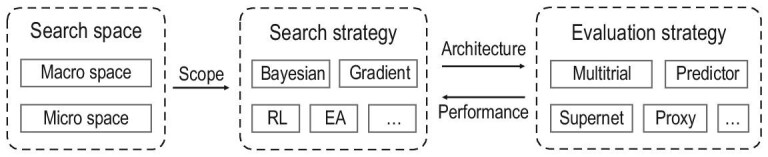
The three key aspects of NAS: search space, search strategy and evaluation strategy.

This paper is organized as follows. First, we discuss the recent development in NAS from the perspectives of the search space, search strategy and evaluation strategy. The relationship between these three aspects is illustrated in Fig. 1. Then, we introduce graph NAS (GraphNAS), i.e. NAS on graphs, which is a trending research direction involving the adaption of NAS to structured graph data with complex typologies and properties. Next, we present recent advances regarding tools and benchmarks for both NAS and GraphNAS. Last but not least, we summarize the paper and provide promising future research directions for NAS.

## DEVELOPMENT IN NEURAL ARCHITECTURE SEARCH

NAS aims to discover the optimal architecture given a particular dataset, which can be formulated as


(1) \begin{eqnarray*}
{\begin{array}{c}{\rm {arg}\, \rm {max}}\\
{a \in \mathcal {A}}\end{array}} \text{Performance}\,(a,D),
\end{eqnarray*}

where *a* is the architecture to be searched in a designed architecture search space $\mathcal {A}$, and $\text{Performance}\,(a,D)$ denotes the architecture’s performance on dataset *D*. Generally, NAS consists of three key modules. (1) The *search space*, which defines the architecture components to be searched, e.g. architecture operations, operation connections, etc. A sophisticated search space may introduce suitable inductive bias and simplify the search process. (2) The *search strategy*, which aims to explore the potentially large search space and discover the optimal architectures with as few architecture samples as possible. (3) The *evaluation strategy*, which aims to estimate the architecture’s performance and is then utilized in the searching process.

### Search space

The search space is very important for NAS. A well-designed search space can greatly improve the search cost and performance of the final architecture. A neural architecture is composed of two parts, namely, the operators and their connection modes. The operators can be neural network layers (e.g. convolution, various nonlinear activation functions), complex blocks, (e.g. ConvBNReLU) and simple computations (e.g. addition and multiplication). Besides the choice of operators, the connections between them have a great impact on the performance of the neural architecture. For example, manually designed architectures like ResNet [43] have demonstrated the effectiveness of skip connections. At present, the search spaces in NAS mainly consist of the different choices of operators and the possible ways to connect them.

Zoph and Le [16] designed the first search space for NAS, which is a sequential search space composed of layers, each containing many convolution operators with different kernel sizes, channel numbers and strides. In addition to connecting directly with the upper layer, it also allows skip connections between different layers. Later work by Ma *et al.* [44] attempted to explore more operator options to improve the performance of the architecture, such as the use of channel shuffle operators.

It is well accepted that a sufficiently large search space that covers good architecture choices is important in a successful NAS. However, larger search spaces come with higher search costs, which can be unacceptable in many cases. To tackle this problem, several approaches have been developed for different application scenarios to design good search spaces with acceptable sizes.

#### Cell-based search space

In order to make the searched architecture portable across different datasets, Zoph *et al.* [17] proposed the first cell-based search space, namely, the ‘NASNet search space’. In cell-based search spaces, neural architectures are dissected into a small set of reusable cells, which can be combined in different ways to produce architectures for different datasets. In NASNet, the cells are found by searching on CIFAR-10, while also having great performance when transferred to ImageNet. Subsequential works further focus on the way cells are arranged into whole architectures.

#### Efficient search space

Another hot direction is to design efficient search spaces for resource-constrained scenarios. By carefully designing search spaces with operators suitable for specific use cases, it is possible to save a lot of search costs and improve the quality of final architectures [45,46]. Moreover, such scenarios often have certain requirements for the size of the model. Therefore, some works add the sizes of operators as a part of the search spaces to facilitate the search for more efficient architectures [47]. FBNetV1 [48] proposed a lightweight layer-wise search space for mobile device application scenarios. FBNetV2 [49] added dimension operators to search for the shape of the architecture.

### Search strategy

The search strategy is a critical component of neural architecture search, which aims to explore the potentially large architecture space efficiently [50]. Given a search space, the search strategy faces the exploration-exploitation trade-off that it has to quickly find optimal architectures as well as avoid the local sub-optimality. Based on the way of encoding architectures, the search strategy can be roughly classified into discrete and continuous search, where discrete search adopts hard encodings of architectures in the search process and the output architectures are the final ones, while continuous search adopts soft encodings, e.g. a probability distribution of the architecture components, and the final architecture can be derived via discretization of the soft encodings, e.g. use argmax.

#### Discrete search

A simple solution is *random search* to randomly sample the architectures from the search space, and select the best performing architecture. However, it cannot well exploit the relationship between architectures and their performance to accelerate the search process.

*RL-based NAS* [51] transforms the problem as a sequential decision-making process, where an agent optimizes their reward and improves their behavior by interacting with the environment. Specifically, an architecture is constructed by a sequence of actions, e.g. adding a layer of neural network operations, altering the hidden dimension, etc. The architecture is then evaluated by the evaluation strategy, and the performance results, e.g. accuracy, can be taken as the reward. The process is repeated many times to train the reinforcement learning controller to obtain the optimal distribution of actions based on the data and states, so that it can discover the optimal architecture given an arbitrary dataset. Several representative RL methods have been adopted in RL-based NAS.

Baker *et al.* [52] adopted *Q-learning* in NAS. The actions include adding layers as well as finishing building the architecture and declaring it complete. The early architectures serve as the states, and the trajectories sampled from this space correspond to models that are subsequently trained to determine their validation accuracy. The Q function is updated by employing the experience replay. To balance exploration and exploitation, they employed an $\epsilon$-greedy approach, where random trajectories are chosen with a probability of $\epsilon$. By selecting a trajectory comprising several decision steps, the algorithm eventually reaches a terminal state, and then trains the corresponding model, updating the action-value function, as defined in the Q-learning algorithm.

Zoph and Le [16] optimized the problem with *policy gradient* methods, where a stochastic policy is parameterized by an auto-regressive RNN controller that predicts actions based on prior actions. The RNN controller in their approach sequentially samples layers that are appended to form the final network, by sampling from a probability distribution obtained through a softmax operation. The final network is trained to obtain performance estimation, while the parameters of the controller are updated using the REINFORCE [53] algorithm.

Negrinho and Gordon [54] solved the problem via *Monte Carlo tree search*. By adopting a tree-structured state-action space that can be explored and expanded incrementally, they utilized a UCT [55] algorithm to explore the tree based on the upper confidence bound. Some other works [56,57] extend the solution by introducing surrogate models to accelerate the search process.

*Evolutionary algorithm-based NAS* [58] treats the architecture’s performance as a black-box function, and adopts evolutionary algorithms [59] to discover the best performing architectures, which commonly include the following key components: (1) initialization for generating the initial population, (2) parent selection to choose parents from the population for reproduction, (3) mutation to generate new individuals and (4) survivor selection to select individuals from the population that will survive. Here, the population consists of a pool of individuals, i.e. neural network architectures. The evolutionary process starts from an initialized population, then some fitness functions, e.g. accuracy, are utilized to guide the parent selection, to breed the next generation. The process is repeated iteratively and the last population is expected to be diverse and optimize the fitness function. There are several representative works, such as the following.

Real *et al.* [60] focused on discovering competitive convolutional neural network architectures for image classification with evolutionary algorithms. The initial population is constructed by generating thousands of simplest possible architectures, and *tournament selection* is adopted for parent selection, which first randomly samples several pairs of architectures, and then the superior ones in each pair are retained along with their weights, mutated and trained before being added to the population. The mutations include adding and removing convolutions and skip connections, changing the kernel size, the number of channels, the stride and the learning rate, etc.

Xie and Yuille [61] described their search space with an adjacency matrix, e.g. numbers in the matrix denote the choice between operations, and each architecture can be encoded by the matrix. The method adopts a *cross-over* operation to conduct the mutation, where a pair of architectures has a probability to random swap the bits in their matrix encodings. The fitness function is defined as the difference between its validation accuracy and the minimum accuracy of the population, so that the weakest individual has a survival rate of zero.

Real *et al.* [62] incorporated age in the selection of survivors, i.e. individuals with better performance but that have spent a longer time in the population might also be removed, which adds the regularization term to the objective function so that the searched architectures are expected to have high performance as well as frequently appear in the population.

#### Continuous search

Continuous search relaxes the operation choices of architectures into continuous encodings so that the searching process can be differentiable.

*Gradient-based NAS* optimizes the operation choices by gradient descents. DARTS [32] relaxes the operation choices by mixed operations, where the operation choice is represented as a probability distribution obtained by softmax of the learnable vectors, and its output is the weighted average of the outputs of all operations. Then the authors propose optimizing the model weights and architecture parameters by respectively minimizing the loss on the training dataset and validation dataset with gradient-based optimization methods. At the end of the search process, the mixed operations usually have to be discretized to obtain the final architecture by choosing the operations with maximum probabilities. The drawback is that the mixed operations require keeping all the candidate operations and their internal outputs in the memory, which limits the size of the search space. To tackle the memory issues, SNAS [63] proposes a factorizable distribution to represent the operation choice so that only one path of the super-network is activated for training, avoiding keeping all operations in the memory. To achieve a similar goal, ProxylessNas [64] also adopts a parameterized distribution over the operations and optimizes with a gating mechanism, where each gate chooses the choice of the path based on the learned probability distribution. Xu *et al.* [65] proposed partial channel connections that randomly sample a subset of channels instead of sending all channels into operation selection to save the memory. TangleNAS [66] proposes a strategy to adapt gradient-based approaches for weight-entangled spaces.

*Architecture decoding.* As architectures are continuously encoded, they need a further architecture decoding to obtain the final architecture, in comparison with discrete search. It has been shown that simply decoding the architecture with maximum probability magnitude is sometimes inconsistent and fails to obtain the optimal architecture [67]. A classic group of methods [68–70] tackle the issue with progressive search space shrinking that gradually prunes out weak operations and connections during the search process to reduce the performance gap caused by the discretization. Wang *et al.* [71] evaluated the operation strength by its contribution to the super-network’s performance, which is estimated by the performance drop after perturbing the operation. Similarly, Xiao *et al.* [72] estimated the operation contribution by Sharley values. Ye *et al.* [73] added an extra $\beta$ decay loss to alleviate the inconsistency problem by regulating the search process.

### Evaluation strategy

The evaluation strategy estimates the architecture’s performance, which includes its expressiveness and generalization abilities [74].

A brute-force solution, as adopted in the *multitrial* search, is to simply train the architecture from scratch on training data and obtain the validation results as the estimated performance [16]. However, this solution is extremely computationally expensive, limiting its usage in practice.

The *weight-sharing mechanism* [75] has been commonly adopted in NAS literature to speed up the performance evaluation of architectures. The idea is to enable the sharing of weights for all architecture candidates so that the training time from scratch can be saved. This technique can be both adopted in discrete search [31] and continuous search [32]. In *one-shot NAS*, a super-network is designed to be trained only once during the search process, and all architecture candidates are viewed as sub-networks of the super-network. In this way, an architecture can be quickly evaluated by selecting the according operation paths and their weights in the super-network. Although the technique can reduce the search time from thousands of GPU days to less than one GPU day [32], it is well known to suffer from inconsistency issues. Given that the weights of sub-networks are highly entangled in the super-network, the training might be severely biased, leading to inaccurate performance estimation [76]. BigNAS [77] finds that the training might be biased to smaller architectures as they have fewer parameters and are easier to converge faster, leading to underestimation of big models. To tackle the issues, they propose a sandwich rule that enforces that the architecture samples should include the biggest and smallest models, to alleviate the training bias with regard to the network size. FairNAS [76] proposes to take expectation fairness and strict fairness into consideration and ensures equal optimization opportunities for all architecture candidates to alleviate overestimation and underestimation. Zhao *et al.* [78] tackled the problem from the perspective of super-networks, where they used multiple super-networks, with each super-network covering different regions of the search space to alleviate the performance approximation gap.

*Predictor-based methods.* The weight-sharing mechanism still needs time training. Currently, there exists a series of NAS tabular benchmarks [29,39–41,79] that documents the performance of all architecture candidates, which can be exploited to train the predictors [80] to predict the architecture’s performance. ChamNet [81] adopts the Gaussian process with Bayesian optimization and builds predictors to predict the latency and performance of the architectures. MetaQNN [82] proposes to predict the architecture’s performance using features from network architectures, hyperparameters and learning curve data. SemiNAS [83] trains an accuracy predictor with a small set of architecture-accuracy data pairs and the predictor is further improved in the search process with newly estimated architectures.

*Zero-shot methods*. To further accelerate the evaluation, zero-shot methods [84] estimate the models’ performance based on specially designed metrics and avoid the cost of training. ZenNAS [85] ranks the architectures by the proposed Zen score that is shown to represent the network expressivity and shows a positive correlation with model accuracy. The calculation of the scores is fast and only takes a few forward inferences through a randomly initialized network without training. NASWOT [86] measures the network’s trained performance by examining the overlap of activations between data points in untrained networks.

*Self-supervised methods*. In some areas where labels are scare or even unavailable, the evaluation of architectures is difficult since fewer labels may result in inaccurate performance estimation. Some NAS methods replace supervised labels with self-supervised loss during the search process [87–91]. Another approach involves designing specialized metrics that do not rely on labels as proxies for estimating model performance. UnNAS [92] employs pretext tasks such as image rotation, coloring images and solving puzzles. Zhang *et al.* [93] trained the model with randomly generated labels, and utilized the convergence speed as the evaluation metric.

## GRAPH NEURAL ARCHITECTURE SEARCH

Besides data in Euclidean space like images and natural languages that are commonly studied in NAS, graph data that are non-Euclidean data are ubiquitous and can model the complex relationships between objects. Graph neural networks (GNNs) [94] are state-of-the-art models for processing graph data. To automate the architectures of GNNs, GraphNAS has received wide attention recently [28]. In this section, we review the advancements in GraphNAS. Since the performance estimation strategy of GraphNAS is similar to previous works, we mainly focus on reviewing the search space and search strategy.

Generally speaking, the differences between general NAS and GraphNAS primarily stem from their target data types, search spaces and architectural components. General NAS aims to optimize neural network architectures for a wide array of data, including images, videos, text and tabular data, by exploring a broad search space that includes various layer types and configurations to capture spatial or sequential patterns. In contrast, GraphNAS is specifically designed for graph-structured data, focusing on selecting and configuring components like graph convolutional layers, aggregation functions and neighborhood sampling strategies to effectively capture the relational and topological properties inherent in graphs. While both approaches face challenges like large search spaces and computational costs, GraphNAS additionally addresses complexities unique to graph data, such as varying graph sizes and sparse connectivity. Consequently, the search algorithms and evaluation metrics are also tailored to the specific needs of their respective data types, with GraphNAS requiring specialized techniques to handle the intricacies of graph neural networks.

### Notation and preliminaries

First, we briefly introduce graph data and GNNs. Consider a graph $\mathcal {G} = ( \mathcal {V},\mathcal {E})$, where $\mathcal {V} = \lbrace v_1,v_2,\dots ,v_{|\mathcal {V}|}\rbrace$ denotes the node set and $\mathcal {E} \subseteq \mathcal {V} \times \mathcal {V}$ denotes the edge set. The neighborhood of node $v_i$ is given by $\mathcal {N}(i)= \lbrace v_j:(v_i,v_j) \in \mathcal {E}\rbrace$. The node features are denoted by $\mathbf {F}\in \mathbb {R}^{|\mathcal {V} | \times f}$, where *f* is the number of features. Most current GNNs follow a message-passing framework [95], i.e. nodes aggregate messages from their neighborhoods to update their representations, which is formulated as


(2) \begin{eqnarray*}
\mathbf {m}^{(l)}_i = \text{AGG}^{(l)}\big(\big\lbrace a_{ij}^{(l)} \mathbf {W}^{(l)} \mathbf {h}^{(l)}_i \text{ for all } j \in \mathcal {N}(i) \big\rbrace \big ),
\end{eqnarray*}

(3) \begin{eqnarray*}
\mathbf {h}^{(l+1)}_i = \sigma \big(\text{COMBINE}^{(l)}\big[ \mathbf {m}^{(l)}_i, \mathbf {h}^{(l)}_i\big] \big),
\end{eqnarray*}

where $\mathbf {h}^{(l)}_i$ denotes the node representation of node $v_i$ in the $l{\rm th}$ layer, $\mathbf {m}^{(l)}$ is the message for node $v_i$, $\text{AGG}^{(l)}(\cdot )$ is the aggregation function, $a_{ij}^{(l)}$ denotes the weights from node $v_j$ to node $v_i$, $\text{COMBINE}^{(l)}(\cdot )$ is the combining function, $\mathbf {W}^{(l)}$ represents the learnable weights and $\sigma (\cdot )$ is an activation function. The node representation is typically initialized as the node features $\mathbf {H}^{(0)} = \mathbf {F}$. Therefore, the final representation is obtained after *L* message-passing layers, resulting in $\mathbf {H} = \mathbf {H}^{(L)}$. To derive the graph-level representation, pooling methods are applied to the node representations


(4) \begin{eqnarray*}
\mathbf {h}_{\mathcal {G}} = \text{POOL}(\mathbf {H}),
\end{eqnarray*}

i.e. $\mathbf {h}_{\mathcal {G}}$ is the representation of $\mathcal {G}$.

### Search space

Since the building blocks of GNNs are distinct from those of other classical deep learning models, e.g. CNNs or RNNs, the search space of GNNs needs to be specifically designed, which can be mainly divided into the following three categories: micro search space, macro search space and pooling functions.

#### Micro search space

Based on the message-passing framework shown in equation (2), the micro search space defines the mechanism by which nodes exchange messages with each other in each layer. A commonly adopted micro search space [96,97] comprises the following components.

- Aggregation function $\text{AGG}(\cdot )$: SUM, MEAN, MAX and MLP.
- Aggregation weights $a_{ij}$: typical choices are shown in Table 1.
- Combining function $\text{COMBINE}(\cdot )$: CONCAT, ADD and MLP.
- Number of heads in attentions: 1, 2, 4, 6, 8, 16, etc.
- Dimensionality of $\mathbf {h}^{l}$: 8, 16, 32, 64, 128, 256, 512, etc.
- Non-linear activation function $\sigma (\cdot )$: sigmoid, tanh, ReLU, identity, softplus, leaky ReLU, ReLU6 and ELU.

**Table 1.** A common search space of different types of aggregation weights $a_{ij}$.

Type	Formulation
CONST	$a_{ij}^{\text{const}} = 1$
GCN	$a_{ij}^{\text{gcn}} = {1}/{\sqrt{| \mathcal {N}(i)|| \mathcal {N}(j)| }}$
GAT	$a_{ij}^{\text{gat}} = \text{LeakyReLU} ( \text{ATT} (\mathbf {W}_a[\mathbf {h}_i, \mathbf {h}_j]) )$
SYM-GAT	$a_{ij}^{\text{sym}} = a_{ij}^{\text{gat}}+ a_{ji}^{\text{gat}}$
COS	$a_{ij}^{\text{cos}} = \text{cos}(\mathbf {W}_a \mathbf {h}_i, \mathbf {W}_a \mathbf {h}_j )$
LINEAR	$a_{ij}^{\text{lin}} = \text{tanh}(\text{sum}( \mathbf {W}_a \mathbf {h}_i + \mathbf {W}_a \mathbf {h}_j ) )$
GENE-LINEAR	$a_{ij}^{\text{gene}} = \text{tanh}(\text{sum}( \mathbf {W}_a \mathbf {h}_i + \mathbf {W}_a \mathbf {h}_j ) )\mathbf {W}_{a}^\prime$

However, directly searching through all these components leads to thousands of possible choices within a single message-passing layer. Therefore, it is beneficial to prune the search space and focus on a few crucial components, leveraging applications or domain knowledge to guide this searching process [98].

#### Macro search space

Similar to other neural networks, one GNN layer does not necessarily solely use its previous layer as the input. These more complicated connectivity patterns between layers, such as residual connections and dense connections [99,100], form the macro search space. Formally, the macro search space can be formulated as


(5) \begin{eqnarray*}
\mathbf {H}^{(l)} = \sum _{j < l} \mathcal {F}_{jl} (\mathbf {H}^{(j)}),
\end{eqnarray*}

where $\mathcal {F}_{jl}(\cdot )$ can be the message-passing layer in equation (2), ZERO (i.e. not connecting), IDENTITY or an MLP.

#### Pooling search space

Pooling search space aims to automate the pooling function in equation (4). For example, Jiang *et al.* [101] proposed the following pooling search space.

- Row-wise sum/mean/maximum:
  \begin{eqnarray*}
\mathbf {h}_{\mathcal {G}} &=&\text{POOL}(\mathbf {H})\\
&=& \mathcal {F}_{\text{pool}}(\lbrace \mathbf {H}_{v,:} \text{ for all } v \in \mathcal {V} \rbrace )
\end{eqnarray*}
  with $\mathcal {F}_{\text{pool}}(\cdot )$ indicating the sum, mean or maximum. Therefore, $\mathbf {h}_{\mathcal {G}} \in \mathbb {R}^d$.
- Column-wise sum/mean/maximum:
  \begin{eqnarray*}
\mathbf {h}_{\mathcal {G}} &=&\text{POOL}(\mathbf {H})\\
&=& \mathcal {F}_{\text{pool}}(\lbrace \mathbf {H}_{:,i} \text{ for all } 1 \le i \le d \rbrace )
\end{eqnarray*}
  with $\mathcal {F}_{\text{pool}}(\cdot )$ indicating the sum, mean or maximum. Therefore, $\mathbf {h}_{\mathcal {G}} \in \mathbb {R}^{| \mathcal {V}|}$.
- Attention pooling:
  \begin{eqnarray*}
\mathbf {h}_{\mathcal {G}} &=& \sum _{v=1}^{| \mathcal {V}|} \sigma ( \mathbf {H}_{v,:}\mathbf {W}_1 + \mathbf {b}_1 )\\
&& \odot (\mathbf {H}_{v,:} \mathbf {W}_2 + \mathbf {b}_2)
\end{eqnarray*}
  with $\mathbf {W}_1,\mathbf {W}_2,\mathbf {b}_1,\mathbf {b}_2$ indicating learnable parameters; the dimensionality of the outputs can be adjusted.
- Attention sum:
  \begin{eqnarray*}
\mathbf {h}_{\mathcal {G}} = \sum _{v=1}^{| \mathcal {V}|} \mathbf {b}_v \mathbf {H}_{v,:}, \mathbf {b} = \text{softmax}(\mathbf {H}\mathbf {W})
\end{eqnarray*}
  with $\mathbf {W}$ denoting learnable parameters. Therefore, $\mathbf {h}_{\mathcal {G}} \in \mathbb {R}^d$.
- Flatten: flat $\mathbf {H}$ into a vector, so $\mathbf {h}_{\mathcal {G}} \in \mathbb {R}^{d| \mathcal {V}|}$.

More advanced methods, e.g. hierarchical pooling [102], could also be incorporated into the search space with tailored designs.

### Search strategy

Early GraphNAS methods directly generalize general search strategies such as reinforcement learning or evolutionary algorithms. To achieve that goal, GNN architectures are usually modeled as a sequence, and methods capable of processing variable-length sequences such as RNNs are adopted as the controller. Differentiable methods can also be directly applied. Though these search strategies are general, they do not consider the explicit characteristics of graphs and thus may not achieve the optimal results. Recent advancements in GraphNAS tackle this problem from different aspects, and we highlight some representative works in the following.

AGNN [97] proposes a reinforced conservative search strategy that utilizes both RNNs and evolutionary algorithms in the controller, which is trained using reinforcement learning. By generating only slightly different architectures, the controller can more efficiently identify well-performing GNNs.

The graph differentiable architecture search model with structure optimization (GASSO) [103] proposes to jointly search GNN architectures and graph structures, aiming to tackle the problem that the input graph data may contain noises. Specifically, GASSO modifies the bi-level optimization of NAS as


\begin{eqnarray*}
\min _{\alpha \in \mathcal {A}} \mathcal {L}_{val}(\mathbf {W}^{*}(\alpha ),\alpha , \mathcal {G}^{*})
\end{eqnarray*}

(6) \begin{eqnarray*}
\text{such that} \quad \mathbf {W}^{*}(\alpha ) &=& \mathop {\arg \min }_{\mathbf {W}} (\mathcal {L}_{train}(\mathbf {W},\alpha )) ,\\
\mathcal {G}^{*} &=& \mathop {\arg \min }_{\mathcal {G}^\prime }\mathcal {L}_{s}(\mathbf {W}^{*}(\alpha ),\alpha , \mathcal {G}^\prime ),\\
\end{eqnarray*}

where $\mathcal {G}^{*}$ indicates the optimized graph structure and $\mathcal {L}_s$ is the smoothing loss function based on the homophily assumption of graphs:


(7) \begin{eqnarray*}
\mathcal {L}_s = \lambda \sum _{i,j} \mathbf {A}^\prime _{i,j} \Vert \mathbf {F}_{i,:} - \mathbf {F}_{j,:}\Vert _2 + \sum _{i,j} (\mathbf {A}^\prime _{i,j} - \mathbf {A}_{i,j})^2.
\end{eqnarray*}

Here $\mathbf {A}$ and $\mathbf {A}^\prime$ represent the adjacency matrix of $\mathcal {G}$ and $\mathcal {G}^\prime$, respectively, and $\lambda$ is a hyper-parameter. By optimizing equation (6), GASSO can simultaneously obtain the best graph structure and GNN architecture in a differentiable manner.

Graph architecture search at scale (GAUSS) [104] further considers the efficiency of searching architectures on large-scale graphs, e.g. graphs with billions of nodes and edges. To reduce computational costs, GAUSS proposes to jointly sample architectures and graphs in training the supernet. To address the potential issues, an architecture peer learning mechanism on the sampled subgraphs and an architecture-important sampling algorithm are proposed. Experimental results show that GAUSS can handle graphs with billions of edges within 1 GPU day.

The graph neural architecture customization with disentangled self-supervised learning (GRACES) [105] improves generalization capabilities in the face of distribution shifts by creating a tailored GNN architecture for each graph instance with an unknown distribution. GRACES utilizes a self-supervised disentangled graph encoder to identify invariant factors within various graph structures. It then employs a prototype-based self-customization strategy to generate the optimal GNN architecture weights in a continuous space for each instance. Additionally, GRACES introduces a customized super-network that shares weights among different architectures to enhance training efficiency. Comprehensive experiments on both synthetic and real-world datasets indicate that the GRACES model can adapt to a variety of graph structures and achieve superior generalization performance in graph classification tasks under distribution shifts [106,107].

The out-of-distribution generalized multimodal GraphNAS (OMG-NAS) method [108] advances the design of multimodal graph neural network (MGNN) architectures by addressing the challenges posed by distribution shifts in multimodal graph data. Unlike traditional MGNAS approaches, OMG-NAS emphasizes the optimization of the MGNN architecture to enhance performance on out-of-distribution data, aiming to mitigate the influence of spurious statistical correlations. To this end, OMG-NAS introduces a multimodal graph representation decorrelation strategy, which aims to refine the MGNN model’s output by iteratively adjusting feature weights and the controlling mechanism to minimize spurious correlations. Additionally, OMG-NAS incorporates a novel global sample weight estimator designed to facilitate the sharing and optimization of sample weights across different architectures. This approach aids in the precise estimation of sample weights for candidate MGNN architectures, thereby promoting the generation of decorrelated multimodal graph representations that focus on capturing the essential predictive relationships between invariant features and target labels. Comprehensive experiments conducted on diverse real-world multimodal graph datasets have validated the effectiveness of OMG-NAS, demonstrating its superior generalization capabilities over state-of-the-art baselines in handling multimodal graph data under distribution shifts.

Data-augmented curriculum GraphNAS (DCGAS) [109] introduces a novel approach to enhancing graph NAS for improved generalization in the face of distribution shifts. This method distinguishes itself by integrating data augmentation with architecture customization to address the limitations of existing graph NAS methods, which struggle with generalization on unseen graph data due to distributional discrepancies. DCGAS employs an innovative embedding-guided data generator, designed to produce a plethora of training graphs that facilitate the architecture’s ability to discern critical structural features of graphs. Moreover, DCGAS innovates with a two-factor uncertainty-based curriculum weighting strategy, which assesses and adjusts the significance of data samples in training, ensuring that the model prioritizes learning from data that most effectively represent real-world distributions. Through a series of rigorous tests on both synthetic and real-world datasets experiencing distribution shifts, DCGAS has demonstrated its capability to learn robust and generalizable mappings, thereby setting new standards for performance compared to existing methodologies.

The robust NAS framework for GNNs (G-RNA) [110] introduces a pioneering strategy to enhance the robustness of GNNs against adversarial attacks, addressing a critical vulnerability in their application to sensitive areas. G-RNA redefines the architecture search space for GNNs by incorporating graph structure mask operations, thereby creating a reservoir of defensive operation choices that pave the way for discovering GNN architectures with heightened defense mechanisms. By instituting a novel robustness metric to steer the architecture search, G-RNA not only facilitates the identification of robust architectures, but also provides a deeper understanding of GNN robustness from an architectural standpoint. This approach allows for a systematic and insightful exploration of GNN designs, focusing on their resilience to adversarial challenges. Rigorous testing on benchmark datasets has demonstrated G-RNA’s capability to significantly surpass traditional robust GNN designs and conventional graph NAS methods, showcasing improvements ranging from 12.1% to 23.4% in adversarial settings, thereby establishing a new benchmark for the design of robust GNN architectures.

Disentangled self-supervised GraphNAS (DSGAS) [111] addresses common scenarios where labeled data are unavailable by identifying optimal architectures that capture various latent graph factors using a self-supervised approach on unlabeled graph data. DSGAS incorporates three specially designed modules: disentangled graph super-networks, self-supervised training with joint architecture-graph disentanglement [112] and contrastive search with architecture augmentations. Experiments conducted on several real-world benchmarks demonstrate that DSGAS achieves state-of-the-art performance compared to existing graph NAS baselines in an unsupervised manner.

Multi-task GraphNAS with task-aware collaboration and curriculum (MTGC$^3$) [113] addresses the challenge of GraphNAS in multitask scenarios by simultaneously identifying optimal architectures for various tasks and learning the collaborative relationships among them. MTGC$^3$ features a structurally diverse supernet that manages multiple architectures and graph structures within a unified framework. This is complemented by a soft task-collaborative module that learns the transferability relationships between tasks. Additionally, MTGC$^3$ employs a task-wise curriculum training strategy that enhances the architecture search process by reweighing the influence of different tasks based on their difficulties. Several experiments demonstrate that MTGC$^3$ achieves state-of-the-art performance in multitask graph scenarios.

Disentangled continual GraphNAS with invariant modularization (GASIM) [114] addresses GraphNAS in continual learning scenarios by continuously searching for optimal architectures while retaining past knowledge. It begins by designing a modular graph architecture super-network with multiple modules to facilitate the search for architectures with specific factor expertise. It then introduces a factor-based task-module router that identifies latent graph factors and directs incoming tasks to the most appropriate architecture module, thereby mitigating the forgetting problem caused by architecture conflicts. Additionally, GASIM incorporates an invariant architecture search mechanism to capture shared knowledge across tasks. Several experiments on real-world benchmarks show that GASIM can achieve state-of-the-art performance compared to baseline methods in continual GraphNAS.

## TOOLS AND BENCHMARKS

### NAS tools

Public libraries are critical to facilitate and advance research and applications of NAS. NAS libraries integrate different search spaces, search strategies and performance evaluation strategies. This different part is modularly implemented and can be freely combined. Users can easily reproduce existing NAS algorithms or extend new ones based on them using the features of the NAS libraries with a small amount of code, which greatly assists NAS researchers and users who wish to use NAS techniques to optimize neural network architectures.

NNI [115] and AutoGL [116] are two open-source NAS libraries. Specifically, NNI automates feature engineering, NAS, hyperparameter tuning and model compression [117] for deep learning. AutoGL (see https://github.com/THUMNLab/AutoGL) pioneers the domain of automated machine learning on graphs by presenting a comprehensive library designed to autonomously determine the optimal machine learning strategy for specific graph-related tasks. Despite the burgeoning interest in AutoML for graphs, existing solutions fall short of providing a holistic toolset. AutoGL emerges as a trailblazer, offering an open-source, user-friendly and adaptable framework. It is the first dedicated framework and library for automated machine learning on graphs, where specialized hyperparameter optimization and NAS algorithms for graphs are included, in addition to classical algorithms. In the recent version update of AutoGL, the library has added several graph algorithm features, such as graph robustness, self-supervised learning and heterogeneous graphs. Users can freely choose to add different features to achieve different functions in the process of using NAS on graphs. The library introduces a meticulously crafted automated machine learning pipeline tailored for graph data, comprising four integral components: auto feature engineering, model training, hyperparameter optimization and auto ensemble. Each module is fortified with a plethora of cutting-edge techniques alongside versatile base classes and APIs, facilitating seamless customization. Moreover, AutoGL enhances its practical applicability by showcasing a series of experimental results, underlining its potential in transforming graph-based machine learning endeavors.

We report the experimental results of AutoGL and some representative baselines on widely adopted node classification benchmarks and graph classification benchmarks. The results are shown in Tables 2 and 3, respectively. We can observe that the results on AutoGL significantly outperform the results on the baselines including GCN, GAT and the GraphSAGE on node classification task and top-K pooling and the GIN on graph classification task, demonstrating the effectiveness of the AutoGL framework.

**Table 2.** The results of node classification.

Model	Cora	CiteSeer	PubMed
GCN	$80.9 \pm 0.7$	$70.9 \pm 0.7$	$78.7 \pm 0.6$
GAT	$82.3 \pm 0.7$	$71.9 \pm 0.6$	$77.9 \pm 0.4$
GraphSAGE	$74.5 \pm 1.8$	$67.2 \pm 0.9$	$76.8 \pm 0.6$
AutoGL	$\mathbf {83.2 \pm 0.6}$	$\mathbf {72.4 \pm 0.6}$	$\mathbf {79.3 \pm 0.4}$

**Table 3.** The results of graph classification.

Model	MUTAG	PROTEINS	IMDB-B
Top-K pooling	$80.8 \pm 7.1$	$69.5 \pm 4.4$	$71.0 \pm 5.5$
GIN	$82.7 \pm 6.9$	$66.5 \pm 3.9$	$69.1 \pm 3.7$
AutoGL	$\mathbf {87.6 \pm 6.0}$	$\mathbf {73.3 \pm 4.4}$	$\mathbf {72.1 \pm 5.0}$

### NAS benchmarks

NAS benchmarks consist of a search space, one or several datasets and a unified training pipeline. NAS benchmarks also provide the performance of all possible architectures in the search space under the unified training pipeline setting. The emergence of NAS benchmarks addresses the following three main issues in NAS research.

- The experimental settings, such as dataset splits, hyperparameter configurations and evaluation protocols vary significantly across different studies. Consequently, this variability makes it challenging to ensure the comparability of experimental results from different methods.
- The randomness of training can lead to different performance results for the same architecture, making the NAS search process difficult to reproduce.
- The performance estimation procedure requires extensive computations and is therefore highly inefficient. The computational demands of NAS research present a significant barrier, rendering it inaccessible to those without substantial computing resources.

Through the NAS benchmarks, different NAS methods can be fairly compared using the unified training protocol. Moreover, NAS methods can get consistent performance estimation to reproduce searching trails. High efficiency of accessing architecture performance enables one to develop new NAS methods conveniently. As a result, NAS benchmarks dramatically boost NAS research.

NAS benchmarks can mainly be divided into tabular benchmarks and surrogate benchmarks. Tabular NAS benchmarks offer pre-computed evaluations for all possible architectures within the search space through a table lookup. In contrast, surrogate benchmarks provide an efficient surrogate function that predicts the performance of all architectures. Tabular benchmarks have better authenticity since the results are from experiments, but running experiments can cost lots of computational resources and potentially limit the size of the search space. Surrogate benchmarks are more efficient, but the quality of the benchmark highly depends on the surrogate function.

So far, lots of different famous benchmarks have been incorporated for NAS. A large part of existing NAS benchmarks focus on computer vision tasks, e.g. NAS-Bench-101 [39], NAS-Bench-201 [40], NATS-Bench [118], NAS-Bench-1shot1 [119], HW-NAS-Bench [120], Surr-NAS-Bench [121], NAS-HPO-Bench-II [122], TransNAS-Bench-101 [123], NAS-Bench-Zero [84], NAS-Bench-x11 [124], and NAS-Bench-360 [125]. Other recent benchmarks also study NAS in a variety of domains, e.g. tabular data (NAS-HPO-Bench [126]), NLP (NAS-Bench-NLP [127] and NAS-Bench-x11 [124]), sequence (NAS-Bench-360 [125]), acoustics (NAS-Bench-ASR [128]) and graph learning (NAS-Bench-graph [129]; see https://github.com/THUMNLab/NAS-Bench-Graph).

## FUTURE DIRECTIONS AND CONCLUSIONS

Recent advancements in the field of large language models (LLMs) have demonstrated their effectiveness in handling graph tasks [130–132] by leveraging their advantages in in-context learning, textual understanding and reasoning capabilities. One promising future direction is to leverage LLMs for GraphNAS, and empower it with more advanced and generalized abilities such as zero-shot learning, in-context learning, etc. This integration would allow GraphNAS to leverage the contextual understanding and reasoning capabilities of LLMs to discover optimal architectural configurations for graph tasks. By exploiting the strengths of both LLMs and GraphNAS, researchers can unlock new possibilities for improving graph-based learning, enabling more efficient training, and enhancing the overall performance and generalization abilities of graph neural networks. Besides, it is worth studying using the coding abilities of LLMs to introduce meaningful variations to code-defining neural network architecture [133]. It also remains to be further explored how to conduct efficient NAS for LLMs for automatically building LLMs with less costs [134].

In addition to graph data, NAS techniques for videos and tabular data is also a promising future research direction, involving automating the design of optimal neural network architectures tailored for specific tasks [135]. For video data, NAS focuses on optimizing architectures that efficiently capture temporal and spatial features, often integrating three-dimensional convolutions and recurrent neural networks to handle the complex dynamics of video frames. In the realm of tabular data, NAS seeks to identify architectures that can effectively manage the diverse and structured nature of tabular inputs, often leveraging fully connected networks, embedding layers and attention mechanisms. These NAS techniques employ various strategies such as reinforcement learning, evolutionary algorithms and gradient-based methods to explore and refine the search space, ultimately improving model performance and efficiency in handling both video and tabular datasets.

The other promising future research direction is multimodal NAS, which is expected to revolutionize how we approach complex, data-rich problems by integrating diverse data types, such as images, text and structured graph data, into a cohesive learning framework. As we move forward, key areas of focus will include developing advanced algorithms that can efficiently navigate the vast search space of possible architectures while effectively fusing multimodal inputs. This necessitates innovations in architecture design to handle the heterogeneity of data types and the development of novel training strategies that can leverage the complementary information contained within different modalities.

In summary, the time complexity of NAS techniques is notably high due to the extensive exploration and evaluation of numerous candidate architectures. This complexity is primarily driven by the size of the search space, the computational cost of training and validating each architecture, and the specific search strategy employed. Reinforcement learning-based NAS can be particularly time-intensive, as it requires iterative training of both the controller and the architectures. Evolutionary algorithms also contribute to high complexity through multiple generations of candidate evaluations. Gradient-based methods, while potentially faster, still face significant computational demands due to backpropagation across a large search space. Advancements such as differentiable architecture search (DARTS) and efficient NAS (ENAS) aim to reduce this complexity by streamlining the search process and leveraging weight-sharing or proxy tasks. Despite these improvements, NAS techniques generally remain computationally expensive, often necessitating substantial computational resources and time to identify optimal architectures. It is interesting to study the efficiency of NAS algorithms.

Lightweight NAS is also an interesting research topic [14,15,50,136–139] that focuses on identifying efficient neural network architectures that balance high performance with low computational cost, making them suitable for deployment on resource-constrained devices such as mobile phones and embedded systems. Unlike traditional NAS, which often results in complex and computationally intensive models, LightNAS emphasizes the creation of models that are compact, have fewer parameters and require less computational power without significantly compromising accuracy. Techniques such as pruning, quantization and knowledge distillation are frequently incorporated into the search process to further reduce the model size and improve inference speed. LightNAS employs strategies like reinforcement learning, evolutionary algorithms and gradient-based methods, but within a constrained search space tailored to prioritize lightweight operations. This approach ensures that the discovered architectures not only perform well, but are also feasible for real-world applications where computational resources and energy efficiency are critical considerations.

Moreover, addressing challenges in scalability, interpretability, robustness, fairness, as well as more training strategies, etc. [47,140–142] will also be crucial, as these systems are deployed across a wide range of applications, from healthcare diagnostics to social network analysis. Ultimately, NAS aims to create a new paradigm for deep learning systems, capable of understanding and analyzing the complex, interconnected data that mirror the multifaceted nature of the real world.
